# PCR-based genotyping assays to detect germline *APC* variant associated with hereditary gastrointestinal polyposis in Jack Russell terriers

**DOI:** 10.1186/s12917-020-02731-7

**Published:** 2021-01-18

**Authors:** Kyoko Yoshizaki, Akihiro Hirata, Hiroyuki Matsushita, Naohito Nishii, Mifumi Kawabe, Takashi Mori, Hiroki Sakai

**Affiliations:** 1 Laboratory of Veterinary Pathology, Joint Department of Veterinary Medicine, Faculty of Applied Biological Sciences, Gifu University, 1-1 Yanagido, Gifu, 501-1193 Japan; 2 Laboratory of Veterinary Internal Medicine, Joint Department of Veterinary Medicine, Faculty of Applied Biological Sciences, Gifu University, 1-1 Yanagido, Gifu, 501-1193 Japan; 3 Laboratory of Veterinary Clinical Radiology, Joint Department of Veterinary Medicine, Faculty of Applied Biological Sciences, Gifu University, 1-1 Yanagido, Gifu, 501-1193 Japan; 4 Laboratory of Veterinary Clinical Oncology, Joint Department of Veterinary Medicine, Faculty of Applied Biological Sciences, Gifu University, 1-1 Yanagido, Gifu, 501-1193 Japan; 5 Center for Highly Advanced Integration of Nano and Life Sciences, Gifu University (G-CHAIN), 1-1 Yanagido, Gifu, 501-1193 Japan

**Keywords:** Hereditary gastrointestinal polyposis, Jack Russell terrier, Genetic testing, Diagnosis

## Abstract

**Background:**

The prevalence of gastrointestinal (GI) neoplastic polyps in Jack Russell terriers (JRTs) has increased in Japan since the late 2000s. Recently, we demonstrated that JRTs with GI polyps harbor identical germline variant in the *APC* gene (c.[462_463delinsTT]) in the heterozygous state. Thus, this disease is an autosomal dominant hereditary disorder. Although the affected JRTs have distinct features, such as the development of multiple GI polyps and an early age of disease onset, genetic testing is indispensable for a definitive diagnosis. Here, polymerase chain reaction (PCR)-based assays capable of detecting germline *APC* variant were designed and validated using synthetic wild-type and mutant DNAs and genomic DNAs from carrier and non-carrier dogs.

**Result:**

First, the PCR-restriction fragment length polymorphism (PCR-RFLP) assay was developed by taking advantage of the germline *APC* variant creating a new restriction site for *Mse*I. In the PCR-RFLP assay, the 156-bp region containing the variant site was amplified by PCR and subsequently digested with *Mse*I, yielding diagnostic 51 and 58 bp fragments from the mutant allele and allowing determination of the *APC* genotypes. It was possible to determine the genotypes using genomic DNA extracted from the peripheral blood, buccal swab, or formalin-fixed paraffin-embedded tissue. Next, a TaqMan duplex real-time PCR assay was developed, where a 78-bp region flanking the variant was amplified in the presence of wild-type allele- and mutant allele-specific fluorescent probes. Using blood-derived DNA, altogether 40 cycles of PCR amplification determined the *APC* genotypes of all examined samples by measuring the fluorescence intensities. Importantly, false-positive and false-negative errors were never detected in both assays.

**Conclusion:**

In this study, we developed highly reliable genetic tests for hereditary GI polyposis in JRTs, providing accurate assessment of the presence of the causative germline *APC* variant. The genotyping assays could contribute to the diagnosis and prevention of hereditary GI polyposis in dogs.

**Supplementary Information:**

The online version contains supplementary material available at 10.1186/s12917-020-02731-7.

## Background

Recent advances in clinical veterinary medicine and molecular genetics, including availability of the complete canine genome sequence [1], have led to the discovery of canine and feline hereditary disorders [2–4]. The cases of gastrointestinal (GI) neoplastic polyps in Jack Russell terriers (JRTs) have increased in Japan since the late 2000s, with the suspicion of it being a novel hereditary disorder [5]. Recently, we demonstrated that JRTs with GI polyps harbor identical germline variant in the *APC* gene, c.[462_463delinsTT] (OMIA ID: 001916–9615), in the heterozygous state. Thus, this is an autosomal dominant hereditary disorder [6]. The solitary and multiple polyps were found in either one or both the stomach and colorectum, with a predilection for the gastric antrum and rectum, in the affected dogs, and most of the GI lesions were histopathologically diagnosed as adenomas or adenocarcinomas [6]. This cancer-prone disease could possibly be a canine counterpart of attenuated familial adenomatous polyposis (FAP) in humans [7].

The following features could provide clinical veterinarians with indications to diagnose hereditary GI polyposis in JRTs: (1) multiple polyps in the stomach and/or colorectum, (2) early onset of GI polyposis, and (3) repeated recurrence [6]. However, as observed in a recent study, this hereditary disease can present as a solitary GI polyp [6], with the lesion distribution similar to those of sporadic GI cancers [6, 8–10]. Although onset at a young age is a distinctive feature, initial GI lesions can arise at a wide range of ages, even after 10 years in JRTs with germline *APC* variant [6]. These findings indicate the necessity of genetic testing for definitive diagnosis of hereditary GI polyposis. Furthermore, JRTs with hereditary GI cancers had a longer survival time than sporadic cases but were at a higher risk for disease recurrence [6], emphasizing the need for differential diagnosis for formulating the treatment and follow-up.

Recently, genetic testing for hereditary disorders is well-established for the diagnosis of affected animals and the prediction of future risks for breed-specific diseases in several breeds [2–4]. Since hereditary GI polyposis is an adult-onset disease [6], future risk assessment is important for early detection of the onset. Furthermore, genetic testing is indispensable to prevent the spread of canine hereditary disorders [2, 3, 11]. Hereditary GI polyposis is spreading among JRTs in Japan since the 2000s, and thus, excluding the carriers of the pathogenic variant from the breeding population is necessary as early as possible.

In a recent study, we discovered a previously unknown disease-causing variant in the *APC* gene using PCR-direct sequencing [6]. Once the disease-causing variant is identified, other PCR-based genetic testing methods such as PCR-restriction fragment length polymorphism (PCR-RFLP) and TaqMan real-time PCR assays can be designed, enabling rapid detection of the target variant in any standard laboratory. These assays are available for the detection of single-base changes, such as point mutations and single nucleotide polymorphisms (SNPs), and used as genotyping assays for canine hereditary diseases [12–17].

In a recent study of hereditary GI polyposis in JRTs, we developed PCR-RFLP and TaqMan duplex real-time PCR assays to investigate the molecular mechanism underlying tumor development [6]. In particular, using these assays, somatic loss of the wild-type *APC* allele was successfully detected in the GI tumors of JRTs with heterozygous germline *APC* variant [6]. These assays would also be applicable to genotyping assays for hereditary GI polyposis, but further validation is required. In this study, we showed that the PCR-RFLP and TaqMan duplex real-time PCR assays were capable of accurately determining the germline *APC* variant status of carrier and non-carrier dogs without any false-positive or false-negative errors.

## Results

### APC variant status of the samples

In this study, genomic DNA samples with heterozygous germline *APC* variant (c.[462_463delinsTT]) were obtained from JRTs with hereditary GI polyposis. These cases were diagnosed as hereditary GI polyposis based on the detection of *APC* variant, and clinical and pathological characteristic findings in our previous study [6]. Samples without *APC* variant were obtained from JRTs with other diseases or laboratory beagles. Genomic DNA samples were extracted from the peripheral blood, buccal swab, or formalin-fixed paraffin-embedded (FFPE) tissue.

**Fig. 1:**
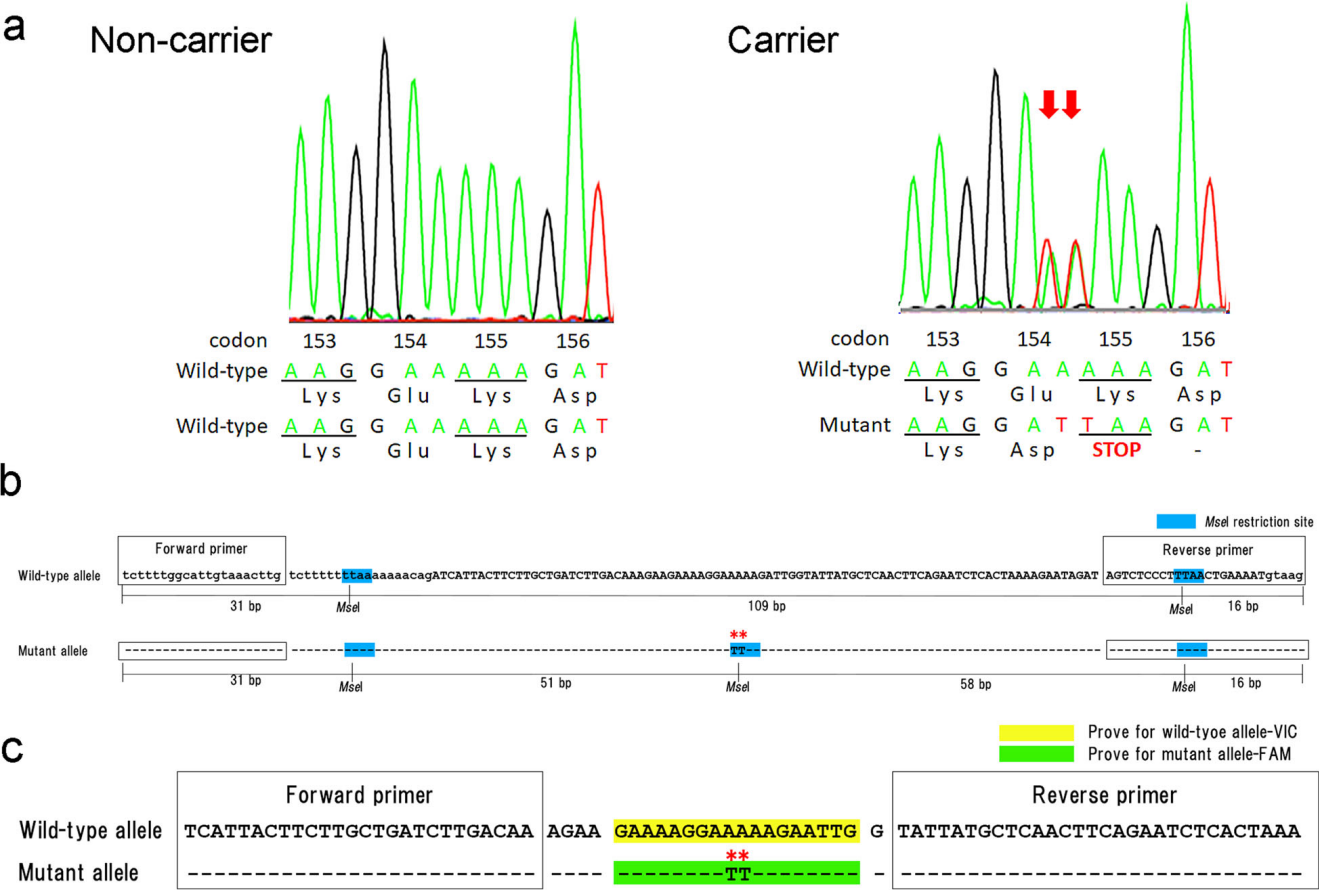
DNA sequence of codons 153–156 in the canine *APC* gene determined by PCR-direct sequencing and schemes of PCR-RFLP and TaqMan duplex real-time PCR assays for the canine *APC* genotyping. **a** PCR-direct sequencing. Representative results of peripheral blood-derived DNAs of JRTs with and without the germline *APC* variants (right and left panels). Note heterozygous deletion-insertion variant at codons 154 and 155 in a carrier dog (arrows). **b** PCR-RFLP assay. Note that, while the 156-bp fragment of the wild-type *APC* allele contains two restriction sites of *Mse*I, there are 3 restriction sites in the fragment of the mutant *APC* allele. Restriction sites of *Mse*I (T/TAA) are highlighted in blue. Asterisks indicate the variant sites. Uppercase and lowercase letters indicate exon 4 and introns, respectively. **c** TaqMan duplex real-time PCR assay. Probes specific for wild-type and mutant alleles are highlighted in yellow and green, respectively. Asterisks indicate variant sites

First, the germline *APC* variant status of all samples used in this study was validated by PCR-direct sequencing. Representative results of PCR-direct sequencing were shown in Fig. 1a. Heterozygous deletion-insertion variant involving two consecutive nucleotides, c.[462_463delinsTT], which spaned codons 154 and 155 of the canine *APC* gene, were detected in all the carrier JRTs, while all non-carrier JRTs and laboratory beagles were homozyous for the wild-type allele.

### PCR-RFLP assay

PCR-RFLP assay was designed by taking advantage of the germline *APC* variant creating a new restriction site for *Mse*I (Fig. 1b). First, the optimal time for enzyme digestion was examined. PCR products amplified from blood-derived DNAs of a carrier and a non-carrier, along with synthetic wild-type and mutant DNAs were digested for 1, 2, 4, 8, 12, and 24 h. The digested fragments of the expected sizes were obtained from each sample after 1 h of digestion (Supplementary Fig. 1), and the shortest digestion time was applied to the subsequent analyses. In addition, consistent with the low star activity of *Mse*I [18], nonspecific digestion was not detected even after 24 h of digestion.

**Fig. 2:**
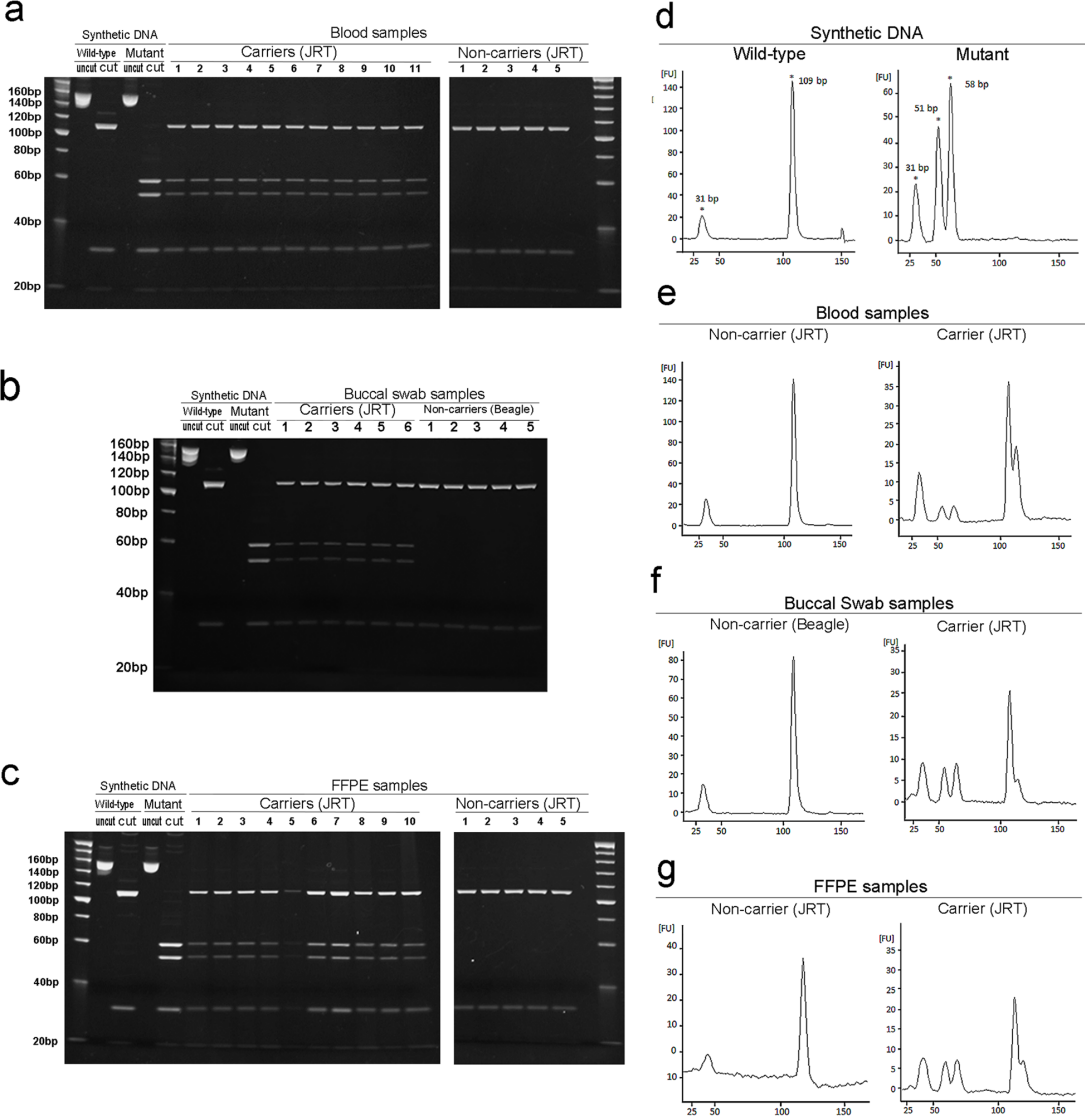
PCR-RFLP assay. **a** Polyacrylamide gel electrophoresis of *Mse*I-digested PCR products amplified from blood samples of carrier and non-carrier JRTs (*n* = 11 and 5, respectively); **b** buccal swab samples of carrier JRTs and laboratory beagles (*n* = 6 and 5, respectively); **c** FFPE tissue samples of carrier and non-carrier JRTs (*n* = 10 and 5, respectively). Representative microfluidic capillary gel electropherograms of *Mse*I-digested PCR products amplified from synthetic wild-type and mutant DNA **d**, blood **e**, buccal swab **f**, and FFPE samples **g** of carrier and non-carrier dogs

The initial PCR produced a single amplicon of 156 bp from all DNA samples, irrespective of the source of the DNA template (data not shown), and subsequent enzymatic digestion yielded distinct fragment patterns, depending on the *APC* genotype. The amplicons from synthetic wild-type and mutant DNAs were cleaved into three fragments of 16, 31, and 109 bp, and four fragments of 16, 31, 51, and 58 bp, respectively (Fig. 2a-c and Supplementary Fig. 1). When analyzing the blood-derived DNA, PCR products from five non-carrier JRTs were cleaved into three fragments and those from 11 carrier JRTs into five fragments, including both wild-type allele-derived 109 bp and mutant allele-derived 51 and 58 bp fragments (Fig. 2a). The same fragmentation patterns were observed in the analyses of the buccal swabs of six carrier JRTs and five non-carrier beagles (Fig. 2b) and FFPE samples of ten carrier and five non-carrier JRTs (Fig. 2c), showing the viability of the assay using other DNA sources.

All digested PCR products were also analyzed by microchip capillary electrophoresis. An electropherogram with different peak patterns was obtained for each genotype. When the synthetic DNAs were analyzed, two peaks at 31 and 109 bp and three peaks at 31, 51, and 58 bp were observed for wild-type and mutant DNA, respectively (Fig. 2d). Representative results of the analysis using genomic DNAs extracted from other sources were shown in Fig. 2e-g. For non-carrier dogs, the peak pattern was the same as that for synthetic wild-type DNA (Fig. 2e-g, left panels). For carrier JRTs, a combined pattern of the wild-type and mutant DNAs was observed, with five peaks at 31, 51, 58, and 109 bp (Fig. 2e-g, right panels). Genotype-specific patterns were observed for all samples obtained from blood (*n* = 5 and 11 for non-carrier and carrier dogs), buccal swab (n = 5 and 6), and FFPE tissues (n = 5 and 10).

### TaqMan duplex real-time PCR assay

**Fig. 3:**
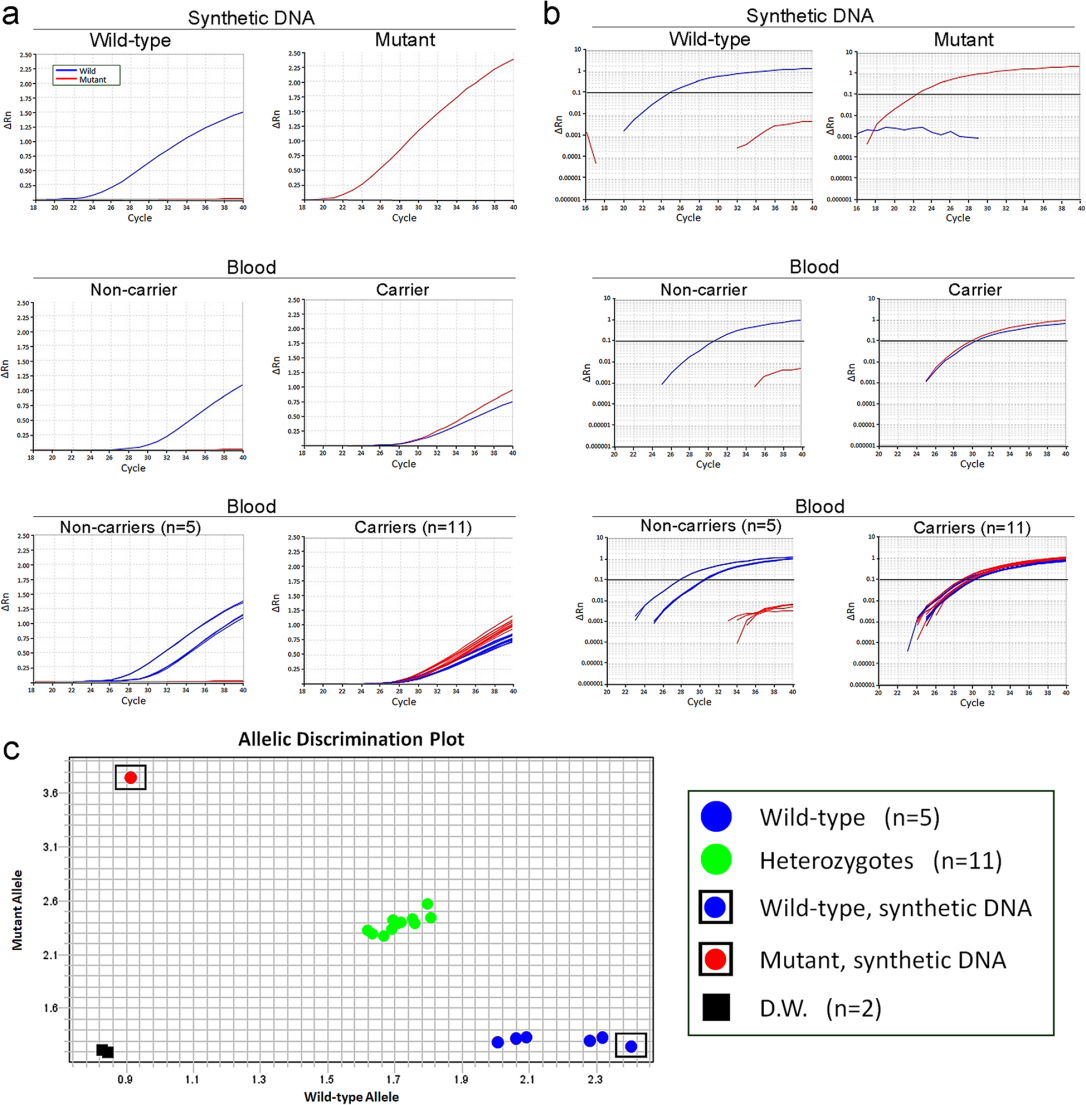
TaqMan duplex real-time PCR assay. **a**, **b** Amplification plots of wild-type (blue curve) and mutant (red curve) *APC* gene copies on synthetic wild-type and mutant DNAs (upper panels) and blood-derived DNAs of carrier or non-carrier JRTs (middle and lower panels). Amplification was plotted as fluorescence intensity (ΔRn value) against cycle numbers and shown on linear (**a**) and log (**b**) scales. The middle panels show representative results of individual cases of carrier or non-carrier dogs and the lower panels summarize the results of all examined cases (n = 11 and 5 for carrier and non-carrier JRTs, respectively). **c** Allelic discrimination plot based on the signal intensity ratio of FAM and VIC at the end points of PCR amplification. Synthetic wild-type and mutant DNAs are located at bottom right and top left corners, respectively, and distilled water used as negative control is at bottom left corner. Non-carrier JRTs are clustered together with the synthetic wild-type DNA at bottom right corner. Carrier JRTs are located near the diagonal line forming a distinct cluster

The real-time PCR assay with TaqMan probes determined the genotypes of all samples. When the synthetic wild-type and mutant DNAs were analyzed, an exponential increase in fluorescence intensity of either VIC or FAM was observed without nonspecific allelic amplification (Fig. 3a and b, top panels). Consistently, when blood-derived DNA samples of 5 non-carrier JRTs were analyzed, only VIC fluorescence intensity increased without detection of FAM fluorescence, even after 40 cycles of amplification (Fig. 3a and b, middle and bottom panels). When the DNA samples of 11 JRTs with the germline *APC* variant were examined, both FAM and VIC fluorescence intensities increased simultaneously and exceeded the baseline level at around 30 cycles (Fig. 3a and b, middle and bottom panels).

Figure 3c shows the allelic discrimination plot constructed based on the signal intensity ratio of FAM and VIC at the end points of PCR amplification. The examined samples were divided into the following three clusters, predicting their genotypes with 100% accuracy: (1) wild-type cluster consisting of synthetic wild-type DNA and five non-carrier dogs, (2) heterozygote cluster of 11 carrier dogs, and (3) the mutant synthetic DNA.

## Discussion

In this study, we developed highly reliable genetic tests for hereditary GI polyposis in JRTs, providing accurate assessment of the presence of the causative germline *APC* variant, c.[462_463delinsTT] [6]. Importantly, false-positive and false-negative errors were never detected in both PCR-RFLP and TaqMan real-time PCR assays, demonstrating the high specificity and sensitivity of the established assays. While PCR-RFLP and TaqMan PCR assays are capable of detecting even a single-base difference, the target *APC* variant is a deletion-insertion variant involving two consecutive nucleotides [6], providing substantial advantage to enhance the specificities of the established assays.

Although the PCR-RFLP assay is one of the most common methods for genotyping, a lack of suitable restriction enzymes hinders its application in some cases. In this study, we successfully developed the PCR-RFLP assay for hereditary GI polyposis by taking advantage of the causative germline *APC* variant creating a new restriction enzyme site for *Mse*I. In the PCR-RFLP assay, *Mse*I digestion yielded diagnostic fragments of 51 and 58 bp from the mutant *APC* allele and differentiated carrier and non-carrier dogs. In this assay, the PCR amplicon contained two restriction sites of *Mse*I besides the variant site; therefore, the 156-bp band disappeared after the digestion regardless of the genotypes, contributing to the prevention of false negative errors by incomplete digestion. Furthermore, the entire process of this assay can be completed within half a day using an ordinary thermal cycler and electrophoresis apparatus, enabling rapid diagnosis in any standard laboratory. In addition, by using the buccal swab as test materials, determination of the genotypes is possible in a less invasive manner. Moreover, when dogs are suspected to have a hereditary disease on histopathological examination of their GI lesions, it is possible to determine their *APC* genotype from FFPE samples without the need for additional blood or swab samples. This approach is also applicable to retrospective studies using archival FFPE samples.

TaqMan duplex real-time PCR assay requires dedicated equipment capable of measuring multiple fluorescences in real time, but this assay enables high-throughput genotyping. Therefore, it is suitable for large-scale molecular epidemiological studies. Another advantage of this assay is determination of genotypes in a single PCR without any additional post-amplification manipulations, thus minimizing the risk of human errors.

The genetic tests developed in this study would provide significant benefits in small animal practice. Clinical veterinarians need to differentiate between hereditary and sporadic GI cancers in dogs for the following reasons. First, JRTs affected with the hereditary disease are much more likely than sporadic cases to have multiple cancers [6]. Second, in the case of the hereditary GI polyposis, the affected dogs can be expected to have a longer survival time than sporadic cases although they have an increased lifelong risk of disease recurrence [6]. The genetic testing enables the differential diagnosis between hereditary and sporadic GI cancers. In addition, JRTs with chronic GI symptoms such as vomiting and bloody stool can be candidates for genetic testing [6]. Considering that endoscopic examination requires general anesthesia in dogs, genetic testing could be an option to predict the occurrence of GI lesions in JRTs. The positive test results strongly support the need for further examinations such as endoscopy. Furthermore, genetic testing would be useful for the assessment of future risk for hereditary GI polyps. Many commercial laboratories offer genetic tests for canine and feline hereditary diseases [4], available to pet owners and veterinarians for assessment of lifelong risk. Hereditary GI polyposis is an adult-onset disease, and initial GI lesions can arise at variable ages, reportedly between 2 to over 10 years [6]. Therefore, knowing the lifelong risk is necessary for early detection and treatment.

Genetic testing is indispensable for preventing the spread of hereditary diseases in dogs [2, 3, 11]. In adult-onset diseases, there is a risk of the carriers of pathogenic variant being unintentionally used for breeding before the disease onset. If a popular sire, such as a dog show champion, is a variant carrier, causative variant can spread rapidly within the breed due to their extensive use for breeding [2]. Our previous retrospective study revealed that the incidence of GI polyposis in JRTs increased since the late 2000s, and that all the affected dogs were born during the first decade of the 2000s [6]. The spread of hereditary GI polyposis among JRTs in Japan might be due to the popular sire effect during this period. Proper genetic screening of sires and dams before breeding would prevent the transmission of germline *APC* variant to future generations. This approach would substantially reduce the future incidence and eventually eradicate this hereditary disease in the future. Therefore, genetic screening should preferably become a routine for animal breeding [2, 4].

The genotyping assays established in this study would certainly facilitate large-scale epidemiological studies. There are no reports of JRTs with hereditary GI polyposis in other countries, except Japan; thus, the germline *APC* variant are possibly prevalent only among JRTs in Japan. However, when examining the pedigree certificates of the JRTs affected by hereditary GI polyposis, some ancestor dogs were introduced from Australia (unpublished data), and thus there is a possibility of the presence of JRTs with the germline *APC* variant in other countries. Furthermore, the prevalence of germline *APC* variant in JRTs in Japan remains to be investigated. Further studies are needed to clarify the prevalence of germline *APC* variant in JRTs in Japan and other countries. Moreover, in future epidemiological studies of healthy JRTs, the carrier dogs could be identified before disease onset, indicating the usefulness of genetic testing for future risk assessment.

## Conclusions

In this study, we established reliable genetic tests for hereditary GI polyposis in JRTs, enabling definitive diagnosis, lifelong risk assessment of this novel disease, and prevention of transmission of germline *APC* variant to future generations. Since dogs affected by this hereditary disease developed multifocal and recurrent GI polyps, development of non-surgical treatment options is desirable. These assays could be incorporated into small veterinary practices in the near future, laying groundwork for the establishment of effective therapy for hereditary GI polyposis.

## Materials and methods

### Samples and DNA extraction

#### Blood samples

Peripheral blood was collected from 11 JRTs with hereditary GI polyposis. Blood samples taken from five JRTs with other diseases (portosystemic shunt, chronic cholecystitis, lymphoma, mast cell tumor and liver tumor) for clinical blood testing at the Gifu University Teaching Animal Hospital served as non-carriers. Genomic DNA was extracted from EDTA-anticoagulated blood samples using the Wizard Genomic DNA Purification Kit (Promega, Madison, WI) or the DNeasy Blood & Tissue Kit (QIAGEN, Venlo, Netherlands) according to the manufacturer’s instructions.

#### Buccal swab samples

Buccal swabs were collected from six JRTs with hereditary GI polyposis and five laboratory beagles using cotton-tipped swabs or 4 N6 FLOQ Swabs™ (COPAN Flock Technologies, Brescia, Italy) and stored at − 20 °C until use. Genomic DNA was extracted using the DNeasy Blood & Tissue Kit (QIAGEN). Briefly, the head of the swab was incubated in a mixture containing 20 μL proteinase K solution (> 600 mAU/mL), 400 μL Buffer A, and 400 μL PBS at 56 °C for 10 min, and the lysate was purified according to the manufacturer’s instructions.

#### FFPE tissue samples

Archival FFPE tissue specimens stored in the Laboratory of Veterinary Pathology at the Gifu University were used. Genomic DNA was extracted from non-neoplastic tissues on FFPE specimens of 15 JRTs, including 10 dogs with hereditary GI polyposis and five with other diseases (chronic gingivitis, fibrous follicular harmartoma, lipoma, fibrous gingival hyperplasia and cystic dilatation of mammary duct), using the QIAamp DNA FFPE Tissue Kit (QIAGEN).

#### Synthetic DNA

Obtaining DNA samples homozygous for the mutant *APC* alleles from dogs is impossible because of embryonic lethality in animals with germline homozygous *APC* variants [19]. To prepare control DNA templates homozygous for wild-type or mutant *APC* alleles, 309-bp DNA fragments were synthesized based on the predicted sequences of the canine *APC* gene (GenBank accession No. NC_006585.3 and XM_014111995.2) (Takara Bio, Shiga, Japan) and cloned into T-Vector pMD20 (Takara Bio). The synthetic DNA fragments contained entire exon 4 (109 bp) with the most proximal 100 bp of both upstream and downstream introns. In the mutant type, in accordance with the germline *APC* variant in the affected JRTs (c.[462_463delinsTT]) [6], adenosine bases at nucleotides 462 and 463 were replaced with thymine bases.

#### PCR-direct sequencing

To validate the *APC* variant status of each sample, PCR-direct sequencing was conducted as previously reported [6]. PCR was performed with TaKaRa EX Taq Hot Start Version (Takara Bio) to amplify a 385-bp fragment containing exon 4 of the canine *APC* gene from the blood and swab samples and a 156-bp fragment containing nucleotides 462 and 463 in exon 4 from the FFPE samples. The primers used are listed in Table 1. After electrophoresis on the 3.0% agarose gel, PCR products were extracted using the QIAquick Gel Extraction Kit (QIAGEN). The purified PCR products were subjected to sequencing analysis using an ABI Prism 3500 Genetic Analyzer (Applied Biosystems, Foster City, CA) with the Big Dye Terminator v3.1 Cycle sequencing Kit (Thermo Fisher Scientific, Waltham, MA).

**Table 1:** Primers and probes used for PCR-based assays for detection of the germline *APC* variant

			Sequence		Product size (bp)
*Primers used for PCR-direct sequencing*					
Primers	sense	5′-	AGTCCCACCTTCAAAAATCC	−3’	385
	antisense	5′-	AACTAAAAATGCAATTATCTTGAATG	−3’	
*Primers used for PCR-RFLP and PCR-directsequencing of FFPE samples*					
Primers	sense	5′-	TCTTTTGGCATTGTGTAAACTTG	−3’	156
	antisense	5′-	CTTACATTTTCAGTTAAAGGGAGACT	−3’	
*Primers and probes used for Taq-Man duplex real-time PCR assay*					
Primers	sense	5′-	TTTAGTGAGATTCTGAAGTTGAGCATAATA	−3’	78
	antisense	5′-	TCATTACTTCTTGCTGATCTTGACAA	−3’	
Probe for Wild-type allele		5′-	(VIC)- CAATCTTTTTCCTTTTC-(MGB)	−3’	
Probe for Mutant allele		5′-	(FAM)-CAATCTTAATCCTTTTC-(MGB)	−3’	

*VIC* 2-chloro-7’-phenyl-1,4-dichloro-6-carboxy-fluorescein, *FAM* 6-carboxyfluorescein, *NFQ* nonfluorescent quencher, *MGB* minor groove binder

### Protocol for PCR-based diagnostic assays

#### PCR-RFLP assay

DNA fragments of 156 bp, containing the variant sites at nucleotides 462 and 463 of the canine *APC* gene, were amplified by PCR using Takara EX Taq Hot Start Version (Takara Bio). PCR reaction mixtures (20 μL) contained 2 μL of 10× Ex Taq buffer (Mg^2+^ plus), 2.5 mM of each dNTP, 0.15 μM of each primer, and 0.75 U of Takara Ex Taq DNA polymerase, and 1 μL of DNA template. The primers used are listed in Table 1. The cycling conditions consisted of an initial denaturation at 94 °C for 5 min, followed by 35 cycles of denaturation at 94 °C for 30 s, annealing at 58 °C for 30 s, extension at 72 °C for 30 s, and a final extension at 72 °C for 5 min. Before enzymatic digestion, 4 μL of the PCR products were analyzed by electrophoresis on 1.5% agarose gel. The remaining PCR products were digested with restriction enzyme *Mse*I (*Rsp*RSII) (Takara Bio) at 60 °C in a 20-μL reaction mixture containing 10 units of *Mse*I, 2 μL of 10× buffer, 2 μL of 0.1% bovine serum albumin, and 15 μL of the product. The digested products were separated by electrophoresis on 15% polyacrylamide gel (SuperSep™ Ace, Wako Pure Chemical Industries, Osaka, Japan). DNA bands were visualized with a UV transilluminator after staining with ethidium bromide (0.5 μg/mL). The digested PCR products were also analyzed by microfluidic capillary gel electrophoresis using an Agilent 2100 bioanalyzer (Agilent Technologies, Santa Clara, CA) with an Agilent DNA 1000 kit (Agilent Technologies).

#### TaqMan duplex real-time PCR assay

Primers were designed to amplify DNA fragments of 78 bp in size, containing the variant sites at nucleotides 462 and 463 of the canine *APC* gene. TaqMan probes were designed to bind specifically to the wild-type or mutant sequences (Thermo Fisher Scientific). The location and sequences of the primers and probes are shown in Fig. 1c and Table 1. The probes were modified with 2-chloro-7′-phenyl-1,4-dichloro-6-carboxy-fluorescein (VIC) or 6-carboxyfluorescein (FAM) at the 5′ end and with a nonfluorescent quencher and minor groove binder (MGB) at the 3′ end. Real-time PCR was carried out in a 50-μL reaction mixture containing TaqMan Genotyping Master Mix (Thermo Fisher Scientific), 0.9 μM of each primer, 0.2 μM of the mutant and wild-type probes, and 10 ng of template DNA. The ABI StepOne Plus system (Applied Biosystems) was used to amplify and quantify the PCR products. The cycling conditions were 95 °C for 10 min, followed by 40 cycles of 95 °C for 15 s and 60 °C for 1 min. Data analysis was conducted using the StepOne software v2.3 (Applied Biosystems).

## Supplementary Information

**Additional file 1: Figure S1**. PCR-RFLP assay conducted to determine the optimal digestion time. Acrylamide gel electrophoresis of *Mse*I-digested PCR products amplified from synthetic wild-type and mutant DNA (A), and blood-derived DNA samples of a carrier and a non-carrier of germline *APC* variant (B). PCR products amplified from each sample were digested with *Mse*I for 0, 1, 2, 4, 8, 12, and 24 h.

## Data Availability

The datasets used and/or analysed during the current study are available from the corresponding author on reasonable request.

## Abbreviations

GI: Gastrointestinal
JRT: Jack Russell terrier
PCR: Polymerase chain reaction
PCR-RFLP: PCR-restriction fragment length polymorphism
FAP: Familial adenomatous polyposis
