# Supplementary material for: PCR-based genotyping assays to detect germline APC variant associated with hereditary gastrointestinal polyposis in Jack Russell terriers
Source: BMC Vet Res. 2021 Jan 18;17:32. doi: 10.1186/s12917-020-02731-7 (PMC7814721; doi:10.1186/s12917-020-02731-7)
Supplement: Supplementary file 1 — Additional file 1: Figure S1. PCR-RFLP assay conducted to determine the optimal digestion time. Acrylamide gel electrophoresis of MseI-digested PCR products amplified from synthetic wild-type and mutant DNA (A), and blood-derived DNA samples of a carrier and a non-carrier of germline APC variant (B). PCR products amplified from each sample were digested with MseI for 0, 1, 2, 4, 8, 12, and 24 h. [file 12917_2020_2731_MOESM1_ESM.pdf]

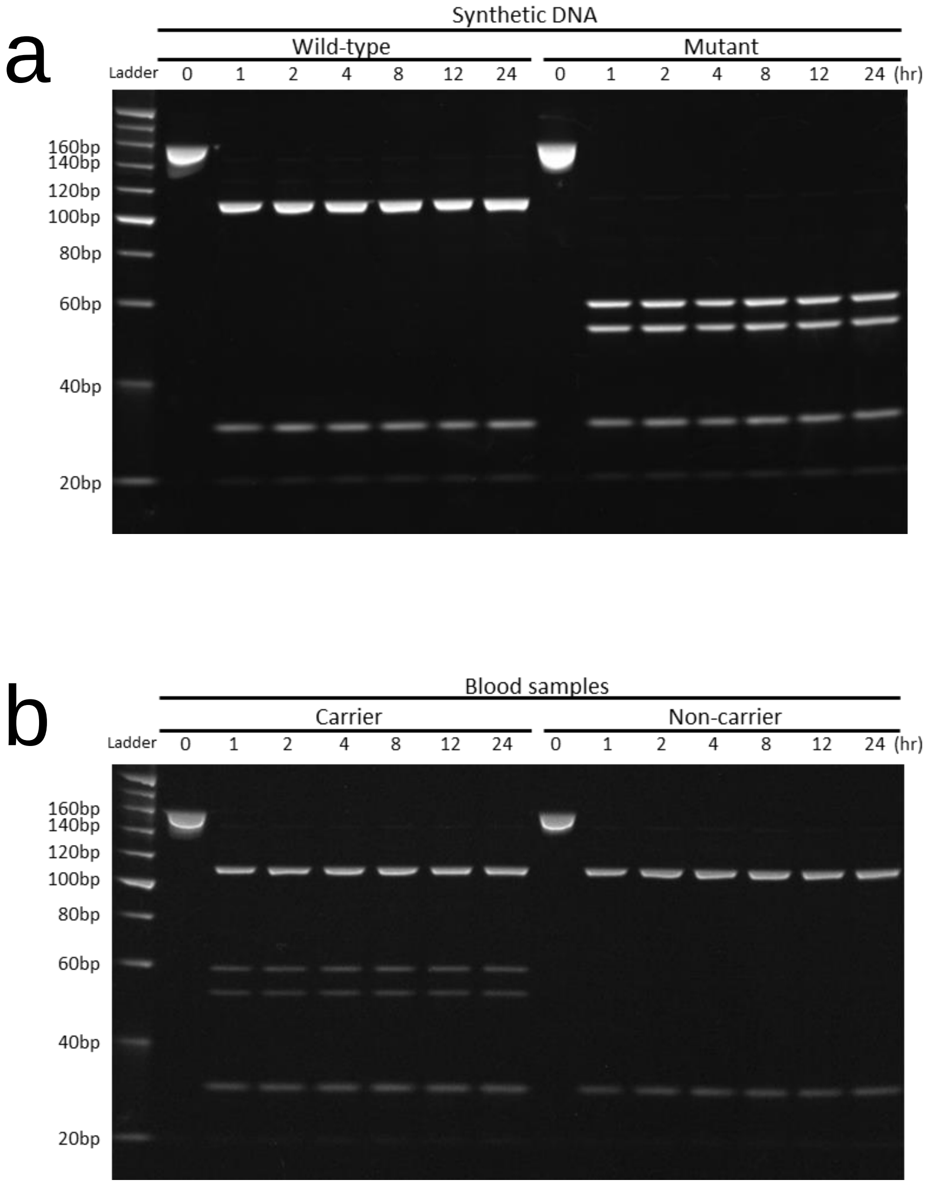

Supplemental Fig. 1
PCR-RFLP assay conducted to determine the optimal digestion time. Acrylamide gel electrophoresis of *Mse*I-digested PCR products amplified from synthetic wild-type and mutant DNA **a**, and blood-derived DNA samples of a carrier and a non-carrier of germline APC variant **b**. PCR products amplified from each sample were digested with *Mse*I for 0, 1, 2, 4, 8, 12, and 24 h.
